# Natural Silk as a Photonics Component: a Study on Its Light Guiding and Nonlinear Optical Properties

**DOI:** 10.1038/srep22358

**Published:** 2016-03-01

**Authors:** Sami Kujala, Anna Mannila, Lasse Karvonen, Khanh Kieu, Zhipei Sun

**Affiliations:** 1 Aalto University, Department of Micro and Nanosciences, PO. Box 13500, FI-00076 Aalto, Finland; 2 Spinverse Ltd., Innopoli 2, Tekniikantie 14, FI-02150 Espoo, Finland; 3 University of Arizona, College of Optical Sciences, 1630 E University Blvd, Tucson, AZ 85721, USA

## Abstract

Silk fibers are expected to become a pathway to biocompatible and bioresorbable waveguides, which could be used to deliver localized optical power for various applications, e.g., optical therapy or imaging inside living tissue. Here, for the first time, the linear and nonlinear optical properties of natural silk fibers have been studied. The waveguiding properties of silk fibroin of largely unprocessed Bombyx mori silkworm silk are assessed using two complementary methods, and found to be on the average 2.8 dB mm^−1^. The waveguide losses of degummed silk are to a large extent due to scattering from debris on fiber surface and helical twisting of the fiber. Nonlinear optical microscopy reveals both configurational defects such as torsional twisting, and strong symmetry breaking at the center of the fiber, which provides potential for various nonlinear applications. Our results show that nonregenerated B. mori silk can be used for delivering optical power over short distances, when the waveguide needs to be biocompatible and bioresorbable, such as embedding the waveguide inside living tissue.

Silkworm silk has been used by the textile industry for thousands of years, due to its excellent physical and biomedical properties, such as flexibility, mechanical strength and most importantly, biocompatibility^1^,^2^,^3^. In particular, the biocompatibility of Bombyx mori silkworm silk has been demonstrated through its use in sutures for several millennia. Silk consists of protein fibers, typically produced by silkworms^3^ and spiders^4^. Recently, silk materials have attracted huge interest for photonics and optoelectronics. Currently existing research on silkworm silk photonics is mainly focused on relatively easily processed, so called regenerated silk. It means silk processed by dissolving purified silk fibers into aqueous solution of LiBr (lithiumbromide) and then casting, spin-coating, printing, or nanoimprinting it to form the desired structures^3^,^5^,^6^,^7^,^8^,^9^,^10^,^11^,^12^,^13^. Regenerated silk has been used to realize various optical elements and photonic devices, such as optical waveguides^4^,^7^, diffraction gratings and microlenses^6^, inverse opals^10^,^12^,^14^, light-emitting transistors^15^, lasers^16^, distributed feedback lasers^17^, and luminescent solar concentrators^18^. Regenerated silk can be functionalized by doping it with e.g., ZnSe and CdTe quantum dots to realize white-light emission^13^, or with azo-benzene sidegroups for optically induced birefringence and holography^19^. Proposals have also been made to use regenerated silk for implantable, bioresorbable silicon electronics devices^20^. Inkjet printed optical waveguides fabricated from regenerated fibroin on glass substrates have been demonstrated to exhibit losses of <1 dB cm^−1^ at 633 nm^7^, which is comparable with polymethyl methacrylate (PMMA) plastic fibers’ loss of ~0.1 dB cm^−1 ^21, silicon strip waveguides (2 ±1) dB cm^−1 ^22, and TiO_2_ strip waveguides (2 ± 1) dB cm^−1 ^23.

However, even though printed silkworm silk waveguides and natural spider silk fibers have been characterized previously^4^,^7^, little research has been conducted on the waveguiding properties of non-regenerated silkworm silk. What makes non-regenerated silk particularly interesting, compared to the more easily utilizable regenerated silk, is that the fibers are naturally organic waveguides without any post-processing. The only processing step required is the degumming process (to be explained later), which does not involve using and subsequently dialyzing metal salts that are byproducts from typical regeneration processes. The only waste product is the sericin protein^3^, which makes silk fibers friendlier for the environment than regenerated silk. Environmental friendliness and biocompatibility, combined with simple fabrication, may be the key arguments for using nonregenerated silk in various medical applications, where the fiber could be embedded in living tissue, and localized optical power delivered thereby directly into the tissue.

In this Work, we present results of our experiments on the waveguiding properties of natural silk, and its nonlinear optical properties for the first time. We assess the loss coefficient of our silk fiber samples using two different methods, the well-known cutback method, and image-based analysis, in which we evaluate the fiber performance using microscope images. We find the loss coefficient to be on the average 2.8 dB mm^−1^, depending on the assessment method and wavelength. Nonlinear optical microscopy reveals both configurational defects such as torsional twisting, and strong symmetry breaking at the center of the fiber, which provide potential for various applications. We will show that the waveguide losses in degummed silk are to large extent due to scattering from debris and torsional twisting of the fiber, both of which originate from the preprocessing and handling of the fibers. The effect of these factors can be minimized, but unfortunately not completely removed during preprocessing.

## Results and Discussion

Silk fibers produced by B. mori and other silkworms contain two protein monofilaments with triangular sections, named brins, embedded in and held together by a water-soluble sericin protein layer. The brins consist of several nanofibril bundles, which in turn consist of fibroin filaments. Sericin is a protein, which is typically removed as it can incite an inflammatory reaction, if silk is used in biological applications. Sericin is also removed in textile applications. This process is known as degumming. The two brins are separated during degumming, and are also called fibroins to distinguish from undegummed silk fiber^3^. In this Work, when discussing about silk fiber, we mean single, degummed brin of B. mori silkworm silk.

**Figure 1:**
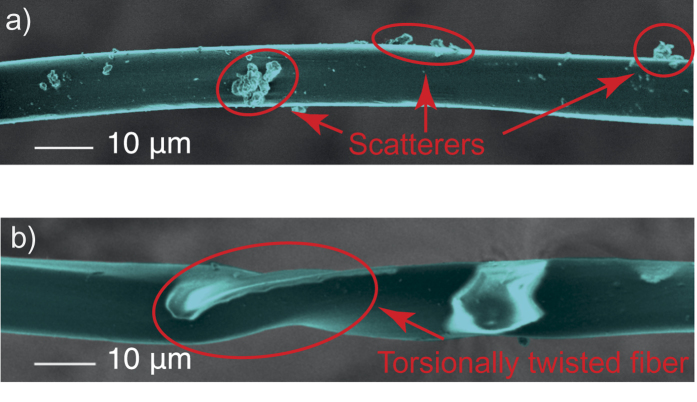
Scanning electron micrographs (SEM) of the silk fibers. Images are pseudocolored for clarity. Part (**a**) shows debris on fiber surface. The largest debris particles are on the order of several µm in diameter. Part (**b**) shows a length of fiber which has been twisted torsionally about its axis.

To study the optical properties of natural degummed silk, we prepared five samples of silk fiber. We assessed fiber structural quality using scanning electron microscopy. Figure 1 shows that the shape of silk fiber is somewhat uniform, although not perfectly circular in shape. Therefore, we expect that it can be used for guiding light. However, two key points need to be noted from the images: there can be debris on the fiber surface, and the fiber can be twisted in a helical fashion about its axis. Both particulate debris on fiber surface and twisting of the fiber can lead to increased attenuation of the guided light through increased scattering losses. It is likely that the amount of particulate scatterers can be taken care of with more thorough preprocessing, during which the washing and rinsing steps could be repeated several times. However, helical twisting is a difficult-to-avoid feature of the silk fiber. It may arise naturally as the silkworm may turn about its axis while spinning the silk fiber, but more importantly, when the fiber is being handled and spooled during its harvesting. The amount of twisting could possibly be reduced by extremely careful harvesting of the fiber from the cocoons. The number of helical twists per unit length can be quantified with for instance second-harmonic imaging or scanning electron microscopy, as will be discussed later.

Silkworm silk fibroin is composed mainly of three amino acids: glycine (G), alanine (A), and serine (S)^3^, which form into semi-crystalline and amorphous sections. The crystalline sections consists mainly of heavy molecular chains (H-chain)^24^, which in turn, are primarily GAGAGS-repeats^25^. The H-chain exists in four forms: amorphous random-coil conformation and three semi-crystalline forms, labeled silk I, silk II^3^, and silk III^26^. Marsh, Corey and Pauling proposed a model^27^, in which the H-chain of silk II forms pleated, anti-parallel *β*-sheets. Silk fibers comprise of protein chains in roughly two-thirds semi-crystalline form and one-third amorphous form^28^, information which can utilized for identifying silkworm silk.

**Figure 2:**
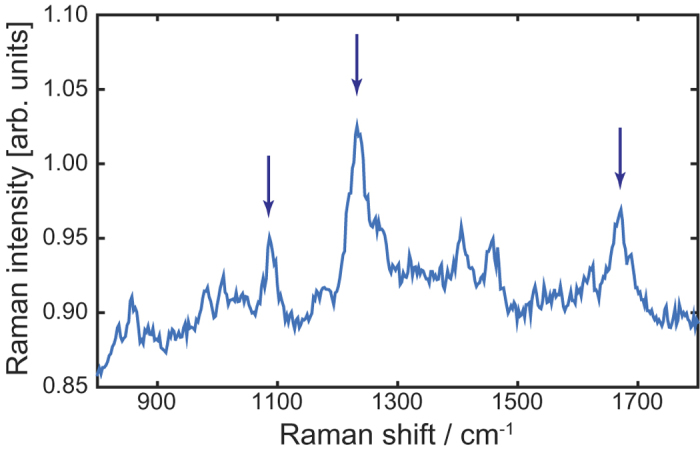
Measured Raman spectrum of the silk fibers used in this Work. Arrows mark the Raman-active bands 1085 cm^−1^, 1232 cm^−1^ and 1667 cm^−1^, which are characteristic to the main proteins in Bombyx mori silk^28^. The remaining features of the spectrum are very similar to the spectrum published in ref. 28.

As the silkworm cocoons were bought from commercial vendors, we wanted to be certain that the obtained degummed silk is truly B. mori silkworm silk. In order to verify the chemical composition of the silk, we characterized the samples using Raman spectroscopy with a confocal Raman microscope operating at 532 nm (WITec Alpha 300 RA). Figure 2 shows the obtained data. Data show prominent Raman-active bands at wavenumbers 1085 cm^−1^, 1232 cm^−1^ and 1667 cm^−1^, which are characteristic to the vibrational modes of the proteins in silk fibroin. The latter two correspond to the *β*-sheets in the silk fiber, i.e., the silk II conformation, and the first one corresponds to random-coil conformation^28^. Also other features of the data are very much similar to those published elsewhere^28^, confirming that the harvested fibers are indeed silk fibroin from B. mori silkworm.

**Figure 3:**
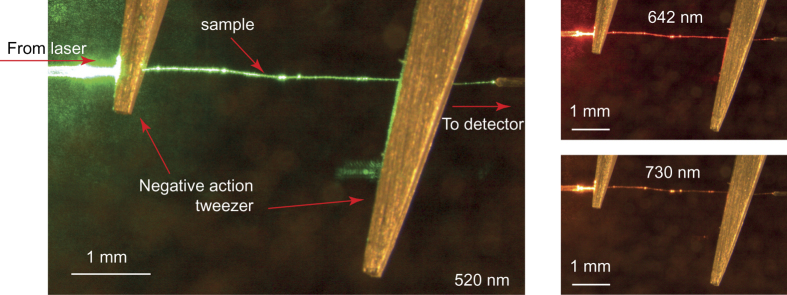
Photographs of the experimental setup. The sample is placed between two negative action tweezers and gently pulled to straighten the fiber. Input light is coupled into the sample and collected with a pickup fiber. The sample is placed between two microlensed optical fibers, which are placed on micromanipulators (not shown) for simple coupling light in and out from the sample fiber.

After verifying the fiber composition, we measured the fibers’ propagation loss using two complementary methods (i.e., the cutback method and image-based analysis method). Detailed discussion about the experimental method is presented in Methods. Typical photographs of the experimental setup are given in Fig. 3. As the figure shows, light is guided inside the silk fiber, from the laser to the detector.

**Figure 4:**
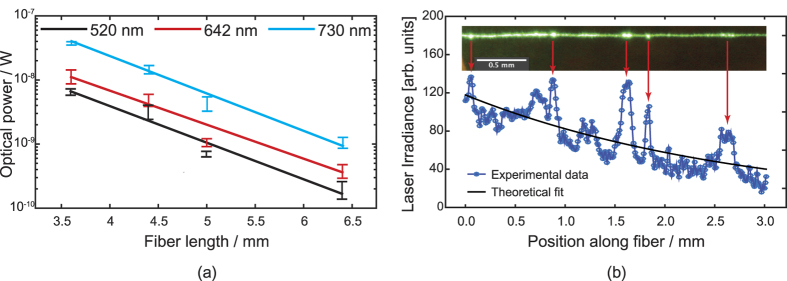
(**a**) Results of cutback experiments on Sample 4 - for other samples, see Supplementary Figure S1. Data points corresponding to different wavelengths are slightly offset in the *x*-direction for clarity. Error bars indicate 25% and 75% percentiles of the data to specify the fluctuations due to, e.g., laser power fluctuation and fiber movements due to air drifts or static electricity. (**b**) Example of silk loss assessment using image-based analysis. Remaining samples have similar features. The inset image is in scale with the data. Inset shows raw image data from Sample 4. Strong scatterers on the fiber surface are most likely debris from preprocessing and handling.

The experimental results of our samples are given in Fig. 4 and Supplementary Figure S1. In the experiments, all wavelengths were measured before cutting the fiber. Each data point corresponds to a data set of thousands of points. Data that was outside the interquartile range of individual data sets was considered outliers and thus discarded. This choice, albeit somewhat arbitrary, gives a good indication of the variability of the data, which is mostly due to fluctuating laser power and input or output coupling as the fibers move easily with drifts of air. Least-squares analysis was used to extract estimates of the loss coefficients.

Figure 4a and Supplementary Figure S1 also show that solid lines fitted to Equation (1) do not go through the mean values of all of the data sets, as they ideally should. It is very likely that a strong scatterer was removed when the fiber was cut during the cutback experiments. To study this effect, we fitted Equation (1) again to Sample 4 cutback data, but this time separately to data points from fiber lengths corresponding to two longest and two shortest lengths. For results, see Supplementary Figure S2 and Supplementary Table S1. We find that the extra fits agree better with the data. This shows that even though the cutback method is very attractive because of its simplicity, when it is applied to relatively unprocessed natural fibers such as silkworm silk or spider silk^4^, the validity of the underlying assumptions becomes questionable, as will be discussed in the Methods section of this Paper. Regardless of these issues, we still expect to obtain at least qualitative and order-of-magnitude results from cutback experiments. Experimental conditions are similar for all samples, and thus meaningful comparisons can be made between the experimental results taken at different wavelengths, to see possible trends.

To complement the cutback technique, we used image-based analysis. It is a particularly attractive method to be used with natural fibers, such as silk fiber, as for instance the fiber does not move during the time it takes to record the image. Figure 4b shows an image of the fiber as it is being illuminated by a 520 nm-laser. The image is unprocessed, except for straightening the image of the fiber with an open source microscopy analysis tool Fiji^29^. The optical image in the inset shows five strong scatterers in Sample 4. They cause the propagating light to leak out from the fiber. A second main advantage of the image-based analysis is then clearly that the scatterers can be observed and their contribution to the losses can be taken into account.

The results for the estimated waveguide loss coefficients for Samples 1–5 are tabulated in Table 1. The cutback method suggests that the loss coefficients lie between 2.27–5.84 dB mm^−1^, with an average value of 3.99 dB mm^−1^. The image-based analysis estimates it to be on the average 1.03 dB mm^−1^, and lying between 0.54–1.55 dB mm^−1^. For comparison, the propagation loss in forcibly reeled, unprocessed Nephila clavipes spider silk has been reported to be (1.05 ± 0.40) dB mm^-1^^4^.

The two methods (i.e., the cutback and the image-based analysis methods) agree on a qualitative level. Both methods yield similar spectral behavior of the loss coefficient: it decreases with increasing wavelength, except for Sample 4, where data suggest that the loss coefficient increases again at 730 nm. Experimental difficulties prevented us from assessing the fiber performance at the telecommunications wavelength of 1.5 μm. However, the wavelength behavior of our samples suggests that the losses should be lower in the infrared region, unless silk fibers contain water, which would increase attenuation.

The reason for the different behavior with respect to wavelength of Sample 4 remains unclear. As the two methods agree qualitatively on loss coefficients’ wavelength dependency, this behavior is unlikely to be a simple measurement error. It is possible that for Samples 1–3 and 5, the losses were mainly due to waveguide composition and shape irregularities, and scatterers whose optical response depends only weakly on the wavelength. For Sample 4, these scatterers’ optical responses may depend more strongly on wavelength.

**Figure 5:**
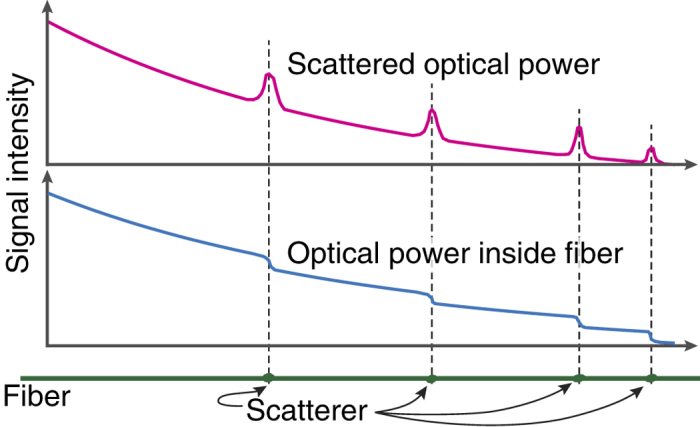
Schematic illustration of explanation for differences between results from cutback technique and image-based analysis.

However, we notice that the cutback results are roughly two times larger than those measured with the image-based analysis. We explain this discrepancy with localized scatterers, as shown in Fig. 1. See Fig. 5 for a schematic illustration. As the light wave propagating inside the fiber (and attenuating according to the Beer-Lambert law) encounters a localized scattering center, a relatively large amount of light escapes the fiber at that given point. Therefore, at that point the optical power inside the fiber drops in a step-like fashion. The remaining optical wave continues its propagation at a lower intensity. When data from this kind of behavior is fitted to the Beer-Lambert law, the obtained loss coefficient will be overestimated, as it will contain contributions from the localized scatterers, in addition to the fiber material and waveguide properties. Thus, the loss coefficient will be larger than if it were only due to material and waveguide properties. At the same time, the scatterers will introduce “hot spots” into the data obtained with the image-based analysis (cf. Fig. 4b). As a result, the loss coefficient becomes underestimated. Therefore, we can conclude that the true material and waveguide property lies somewhere between the values obtained with these two methods.

**Figure 6:**
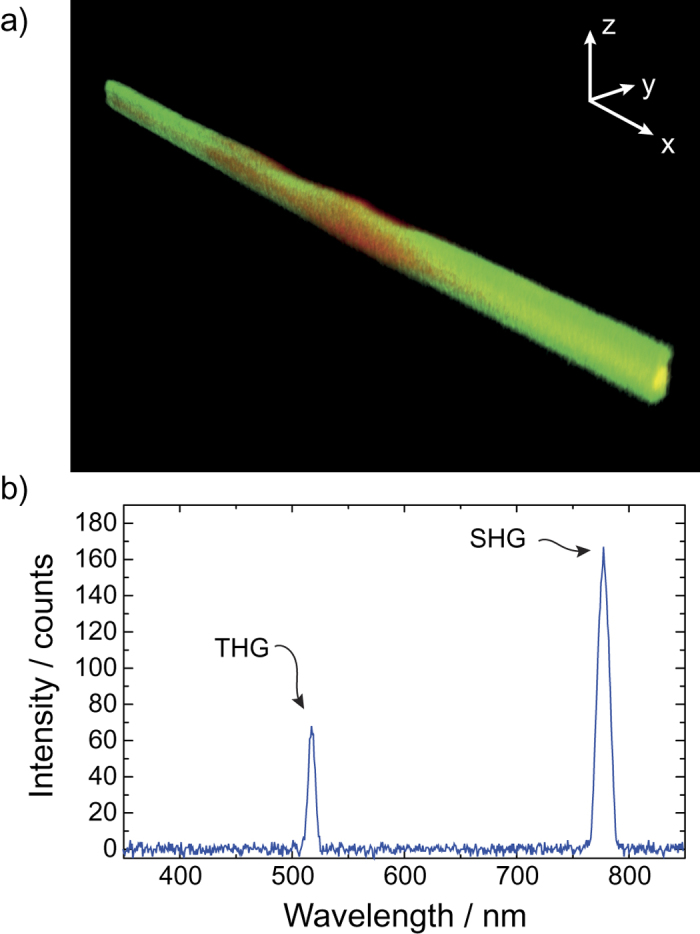
(**a**) Reconstructed image of the silk fiber, as imaged using a multiphoton microscope (1560 nm excitation femtosecond fiber laser). Colors: SHG - red and THG - green. Yellow areas produce both signals. (**b**) Optical spectrum of the measured SH and TH signals indicates that the signals are indeed due to SHG and THG, respectively.

An image of the silk fiber as recorded by a nonlinear microscope is shown in Fig. 6. The red color represents the signal due to second harmonic generation (SHG) and the green represents third harmonic generation (THG). Yellow areas produce both second and third harmonic light. As can be seen from the figure, the third-harmonic signal is generated all over the silk fiber sample. SHG however, is strongest in the middle of the fiber. We attribute this to the fiber being twisted about its axis at this point. The torsional twisting introduces chirality into the fiber structure, and therefore breaks the centrosymmetry which is a requirement for SHG to be possible^30^. Helical twisting is also observed in the scanning electron micrograph (SEM) characterization (Fig. 1b). Our results show that silk-based nonlinear components could be used for various applications (e.g., *in vivo* twisting sensing in tissue).

## Summary

To summarize, we have prepared and characterized the waveguiding properties of B. mori silkworm silk fibers. We determined the value of propagation loss coefficient (2.8 dB mm^−1^ on the average) at three different wavelengths. We found that the propagation loss coefficient decreases with longer wavelength. We showed that the loss coefficient is composed of two primary effects: material and waveguide properties, and localized scatterers (such as debris on fiber surface and helical twisting of the fiber).

Nonlinear optical microscopy reveals both configurational defects such as torsional twisting, and strong symmetry breaking at the center of the fiber, providing potential for various nonlinear applications. Our results demonstrate that nonregenerated silk fibers could become a pathway resulting in biocompatible and bioresorbable waveguides. These waveguides could be used to deliver localized optical power for various applications (e.g, inside living tissue).

The effect of the scatterers can most likely be minimized by careful and thorough degumming process, and careful handling and spooling of the fiber. To our understanding, the relative importance of debris vs. twisting on scattering cannot be separated in a quantitative way by performing cutback experiments or image-based analysis. However, nonlinear optical microscopy and scanning electron microscopy could be used to quantify the relative amount of twists and debris per unit length of the fiber, and thereby gaining more understanding on their relative strength.

## Methods

In order to have degummed silk fiber, which could be used for optical experiments, silkworm cocoons were processed as follows. The cocoons were preprocessed by soaking them in 53–55 °C deionized water in order to loosen the fiber from the cocoon for reeling, and to remove some of the sericin protein. Fiber was harvested from the inner layers of the cocoon and dried. Only the inner layer fiber was used, as the fiber from outer layers of the cocoon is more likely to be damaged from being handled. The remaining of the sericin was degummed by soaking the harvested fibers in 80–86 °C 0.04 mol L^−1^ Na_2_CO_3_ (sodium carbonate) solution for 30 minutes. As a last step, the fibers were rinsed in deionized water and dried overnight. In total five samples were prepared.

### Optical Experiments

In order to assess the applicability of the silk fibers as natural biocompatible waveguides, their waveguiding properties were characterized optically using two different methods. To gain further understanding on their structure, the samples were also imaged with a nonlinear optical microscope.

#### The Cutback Method

The cutback method is a well-known and simple method for determining losses in waveguides such as optical fibers used in telecommunications. In this work, we apply this method to assess the loss coefficient on our silk fibers. The method is based on the well-known Beer-Lambert attenuation law, which assumes that propagation losses along the fiber length vary slowly enough to be considered a constant. The Beer-Lambert law connects the input and output optical powers *P*_0_ and 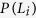, respectively, and the length of the specimen 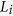 with the loss coefficient *α* as follows:


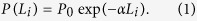


In the cutback method, it is assumed that the input power 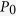 and the input and output coupling efficiencies [omitted in Equation (1)] remain constant. By repeating the cut-and-measure procedure several times, it is possible to extract values for *α* and 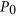 through numerical fitting.

To help mitigate the effect of bending losses with the silk fibers, they were placed between two negative-action metal tweezers, which were gently pulled away from each other to straighten the fiber (cf. Fig. 3). The samples were placed between two microlensed optical fibers (OZ Optics TPMJ-3S1550-8/125-0.4-10-2.5-14-1), one for delivering the excitation, and one for collecting the light coming out from the silk fiber. The optical fibers were mounted on micro positioners to facilitate simple aligning. The excitation was a laser with multiple fiber coupled diode laser sources (Thorlabs MCLS1). The laser sources that were used operate at wavelengths 520 nm, 642 nm and 730 nm. A calibrated silicon photodiode (Ophir PD300-UV-ROHS) was used as the detector of the light emitted from the silk fiber. The lengths of the sample fibers during the cutback experiment were assessed from the images taken for image-based loss assessment, using Fiji.

Its simplicity notwithstanding, the cutback method is not without its challenges, especially when applied to non-rigid natural fibers such as the ones discussed in this Work. Natural silk is not circular in shape and its inner structure can be inhomogeneous. The fiber may become twisted about its axis during its handling and spooling. Moreover, there may also be debris left over from the degumming process, all of which may lead to localized scattering losses. The assumption of constant loss coefficient may not anymore be entirely justified (cf. Fig. 1). Therefore, the cutback technique tends to overestimate the loss coefficient.

#### Image-based Analysis

Images shown in Fig. 3 inspired us to complement the cutback method by attempting to assessing fibers’ performance directly from the microscope images. This method relies on image-based analysis using an established open source microscopy analysis tool Fiji^29^. In this method, it is assumed that the amount of scattered light from the side of the fiber, at any given point, is directly proportional to the irradiance of the wave propagating inside the fiber at that point. Therefore, measuring the scattered light as a function of position along the fiber is expected to provide a quantitative measure of the wave attenuation inside the fiber.

Experimental procedure for the image-based analysis is as follows. The laser is adjusted to couple its light into the silk fiber. Images of the fiber side view are taken. The laser power is adjusted so as to provide strong signal without saturating or damaging the charge-couple device (CCD) array of the camera. The image analysis is done by first setting a correct scale for the image in Fiji, and then extracting the pixel values corresponding to the fiber. Equation 1 is finally fitted to this data to obtain an estimate of the loss coefficient *α*. This method is again a tradeoff between simplicity and accuracy. Simplifying assumptions need to be made: e.g., the response of the CCD array is assumed to be linear, the fiber is assumed to be lying mostly in the object plane of the microscope, and individual pixels of the CCD array are assumed not to be saturated.

#### Nonlinear Optical Microscopy

**Figure 7:**
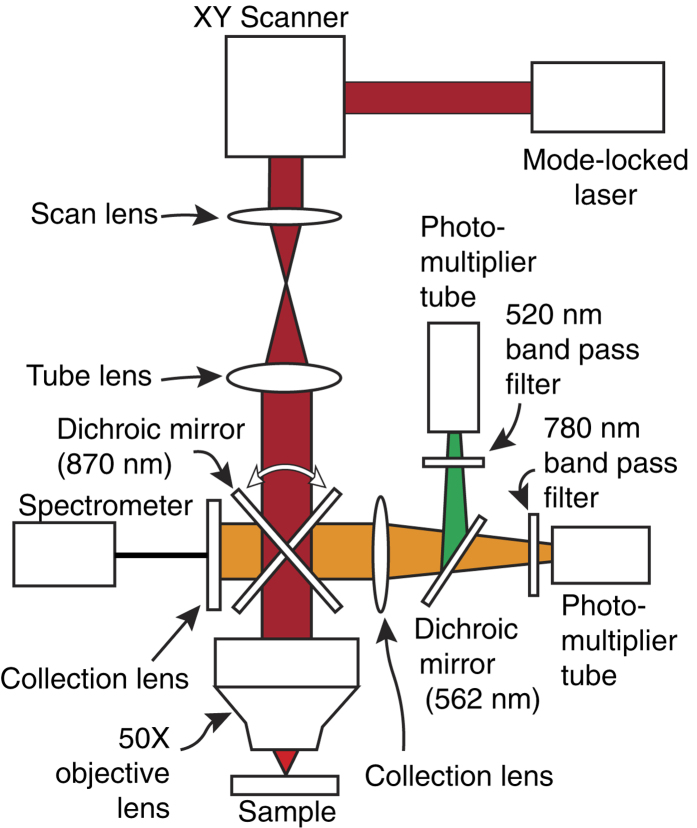
Schematic illustration of multiphoton microscope optical setup.

The nonlinear optical properties of the silk fiber were investigated using a multiphoton microscope, built in-house, which is shown schematically in Fig. 7. The design of the microscope and the experimental procedure were previously reported^31^,^32^,^33^. The light source in the system is an amplified erbium-doped mode-locked fiber laser (center wavelength 1560 nm, average power on the sample 30 mW, repetition rate of 50 MHz, and pulse duration 150 fs). The laser beam is scanned with a 2D galvo mirror system and focused on the sample using a 50× microscope objective (NA 0.8). The diameter of the measured focal spot size is on the order of 1.1 μm. The backscattered second and third harmonic signals generated at each point on the sample are detected using photomultiplier tubes. Narrow band-pass filters are used to select signals due to SHG and THG at central wavelengths 780 nm and 520 nm, respectively. The acquisition of the two channels is simultaneous, making the experimental conditions for both channels exactly identical. A Z-stack image is captured in order to produce a 3D image, which is processed in Fiji. For spectral resolving, the generated light is guided to a spectrometer (Ocean Optics QE PRO-FL).

## Additional Information

**How to cite this article**: Kujala, S. *et al.* Natural Silk as a Photonics Component: a Study on Its Light Guiding and Nonlinear Optical Properties. *Sci. Rep.* **6**, 22358; doi: 10.1038/srep22358 (2016).

## Supplementary Material

Supplementary Information

## Figures and Tables

**Table 1:** Determined loss coefficients in units of dB mm^-1^ as determined with the cutback method and image-based analysis.

Wavelength	Cutback method			Image-based analysis		
	520 nm	642 nm	730 nm	520 nm	642 nm	730 nm
Sample 1	5.15(12)	4.07(16)	3.91(8)	1.34(16)	1.13(22)	0.98(42)
Sample 2	3.67(2)	3.61(3)	2.75(3)	1.50(19)	1.30(11)	—
Sample 3	5.20(5)	3.15(4)	2.27(13)	0.58(5)	—	0.67(23)
Sample 4	5.71(6)	5.31(8)	5.84(3)	1.55(21)	0.54(13)	0.67(23)
Sample 5	2.91(3)	3.40(3)	2.90(3)	—	—	—
Average	4.53	3.91	3.53	1.24	0.99	0.77

Numbers marked with dash (−) mean missing data. Numbers in parenthesis indicate the uncertainty in fitted parameters as given by the fitting routine.
