# Supplementary material for: Natural Silk as a Photonics Component: a Study on Its Light Guiding and Nonlinear Optical Properties
Source: Sci Rep. 2016 Mar 1;6:22358. doi: 10.1038/srep22358 (PMC4772132; doi:10.1038/srep22358)
Supplement: Supplementary Information [file srep22358-s1.pdf]

Natural Silk as a Photonics Component: a Study on Its Light Guiding and Nonlinear Optical Properties

Sami Kujala[1,*], Anna Mannila[1,2], Lasse Karvonen[1], Khanh Kieu[3], and Zhipei Sun[1,*]

[1]Aalto University, Department of Micro and Nanosciences, PO. Box 13500, FI-00076 Aalto, Finland
[2]Spinverse Ltd., Innopoli 2, Tekniikantie 14, FI-02150 Espoo, Finland
[3]University of Arizona, College of Optical Sciences, 1630 E University Blvd, Tucson, AZ 85721, USA
[*]sami.kujala@aalto.fi
[*]zhipei.sun@aalto.fi

ABSTRACT

Supplementary material to complement the main article of details of the obtained data and some additional results.

List of Figures

S1 Results of cutback experiments on Sample 1–3 and 5 2
S2 Results of cutback experiments on Sample 4 3

List of Tables

S1 Determined loss coefficients as determined with the cutback method 4

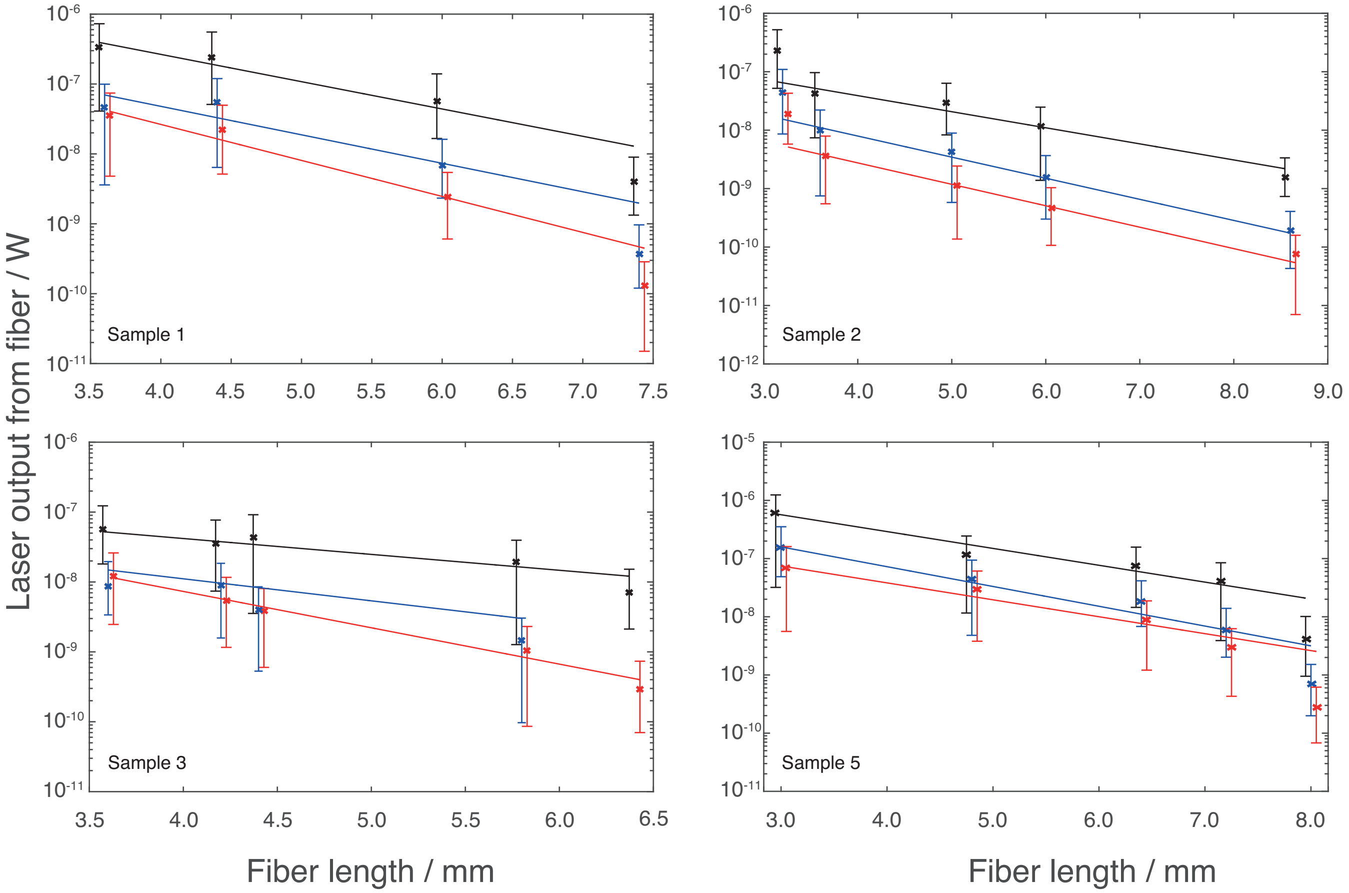

Figure S1. Results of cutback experiments on Sample 1–3 and 5. Again, the error bars indicate 25% and 75% percentiles of the data. Data for 520 nm and 730 nm is slightly offset to right and left, respectively, to improve readability. Lines are fits to the Beer-Lambert law [Equation (1)].

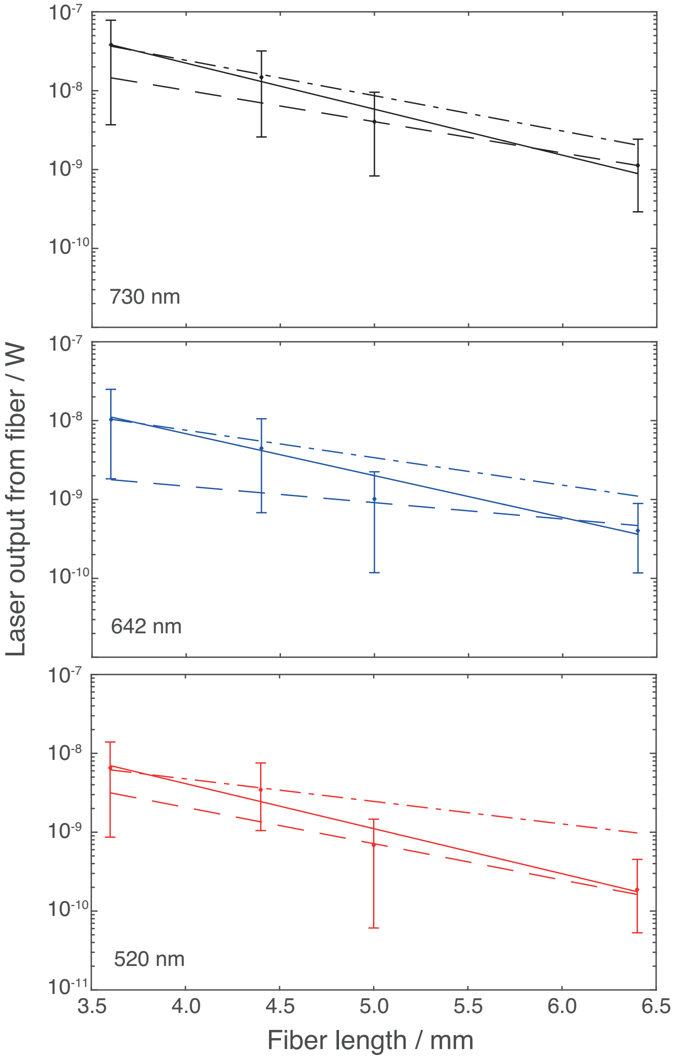

Figure S2. Results of cutback experiments on Sample 4. The error bars indicate 25% and 75% percentiles of the data. Legend: Lines are fits to the Beer-Lambert law [Equation (1)] fitted to; all data points – solid lines, data sets of two shortmost fiber lengths – dash-dot lines, and data sets of two longest fiber lengths – dashed lines.

Table S1. Determined loss coefficients in units of dB mm-1 as determined with the cutback method. Numbers in parenthesis indicate the uncertainty in fitted parameters, as given by the numerical fitting routine.

Wavelength	520 nm	642 nm	730 nm
Sample 4	5.71(6)	5.31(8)	5.84(3)
Sample 4[a]	4.20(5)	4.12(15)	5.58(6)
Sample 4[b]	4.89(7)	2.84(5)	4.78(11)

a) Data sets corresponding to two longest fiber lengths used for fitting; b) Data sets corresponding to two shortest fiber lengths used for fitting.
